# A qualitative analysis of the effectiveness of telehealthcare devices (ii) barriers to uptake of telehealthcare devices

**DOI:** 10.1186/s12913-017-2270-8

**Published:** 2017-07-06

**Authors:** Natasha C. Campling, David G. Pitts, Paul V. Knight, Richard Aspinall

**Affiliations:** 1 Faculty of Health Sciences Building 67, University of Southampton, Southampton, UK; 2 Cranfield Biotechnology Centre, Cranfield University, Bedford, UK; 3 Health and Wellbeing Academy, Anglia Ruskin University, Bishop Hall Lane, Chelmsford, CM1 1SQ UK

**Keywords:** Telecare, Telehealth, Devices, End-users, Barriers, Uptake

## Abstract

**Background:**

Monitoring health and care needs through the use of telehealthcare devices has been proposed to help alleviate funding concerns in a climate of limited budgets. As well as improving cost effectiveness, such an approach could be used to help individuals live at home for longer. In practice however, these devices often go unused. A qualitative study was carried out to determine the barriers to uptake of these devices from both the perspective of the end user and from key players in the healthcare supply chain.

**Methods:**

A qualitative approach was used involving focus groups and interviews. Two UK-based focus groups were held with users and potential users, to assess their views on the wide array of devices available. 27 individuals were involved in the groups, all over the age of 60. Additionally 27 telephone interviews were conducted with key supply chain players to ascertain their views on the barriers to uptake of these devices. A semi-structured interview guide was used. All data were audio-recorded, transcribed verbatim and analysed using a thematic approach.

**Results:**

Users were generally unaware of the wide array of devices available and when shown a selection, were often unclear as to their purpose. The interviews revealed extensive barriers to uptake due to lack of awareness, unfamiliar terminology, complex supply routes and costs, resistance from professionals to device usage and lack of expertise.

**Conclusions:**

Public and professional awareness campaigns are required with appropriate funding mechanisms for users to gain access to devices. The numerous barriers identified require systematically addressing, so that device usage is better promoted, enabling individuals to live at home successfully for longer.

## Background

One of the significant drivers of the telehealthcare industry is the ability to provide cost effective care. A recent report [1] suggested there were three factors contributing to the need for a cost-effective care programme notably; demographic shifts in the population profile towards a population containing more older individuals, a rise in the number of individuals experiencing a significant number of co-morbidities which need to be managed [2] and finally the increasing burden placed on limited healthcare budgets. Telehealth has been seen as a potential solution to these problems. Moreover in an environment where more services are individually tailored, telehealth has been seen as a route to increasing access to care especially for conditions and populations for whom care was limited [3]. As a consequence there have been a number of initiatives introduced to develop this market. Most recent was the launch of Technology Enabled Care Services by NHS England [4]. This had been preceded by the “3millionlives” campaign [5] an initiative developed on the principle that at least three million people with long term conditions and/or social care needs could benefit from the use of telehealthcare and related services. This in turn was set up following the interim results of the Whole Scale Demonstrator programme. This rebranding and re-focussing of initiatives suggests that despite the financial imperative for the introduction of such devices there were and continue to be profound barriers to implementation.

Major barriers to uptake of telemedicine at an infrastructure level have been described previously [1, 6]. In the main these are linked with legal and regulatory considerations mainly associated with the aspects of patient privacy and confidentiality with respect to issues such as data transfer and storage and sharing between healthcare professionals and also their liability when offering telemedicine services. In addition there have been concerns about the technological challenges and the liability of providers in the face of malfunctioning systems [7]. Furthermore there are practical issues to consider associated with access to basic requirements such as adequate wireless broadband which may be limited by geographical reasons. These may appear to some to be second order problems associated with the infrastructure whilst first order problems closer to the device user and associated with product uptake, acceptability and usage were of greater concern.

Many technologies are currently available which can assist in activities of daily living however there are several concerns raised about their uptake by those who would benefit most from them. With this in mind we undertook this study to outline potential users views of current telehealthcare devices in the homecare market, from pendant alarms through to blood pressure monitors and tracking devices and to determine what the barriers were to their uptake.

## Methods

The study utilised focus groups with users and telephone interviews with experts with experience of telehealthcare devices and the telehealthcare sector. Both approaches were carried out after ethical approval.

### Focus group participants and design

The inclusion and exclusion criteria for the focus groups were broad to reduce the potential for bias. Participants for focus groups were recruited from different geographical locations (Bedfordshire and Oxfordshire) and sought through local group organisations. Individuals interested in taking part were contacted and the study outlined further where necessary. If the individual wished to take part consent forms were sent either via post or email. Fifteen participants took part in the Bedfordshire focus group (with no non-attendees). The group was balanced for gender, with eight participants being male and seven female. Their ages ranged between 65 and 84 years of age, four being between 65-69, three between 70-74, five between 75-79 and three from 80-84. In terms of health problems, four stated that they had mobility issues (one used a mobility scooter). One participant was using a pendant alarm (supplied via social services and operated via the local council). The majority (with a sole exception) owned a mobile phone and all were using computers/tablets and email. A number of the group also had access to blood pressure monitors at home, which had been privately purchased. The group in Oxfordshire also saw high attendance with 12 of the 13 participants originally recruited attending. Eleven of the group were female and one male. The age range was wider than with the Bedfordshire group, from 60 to over 85 years of age. Two were aged between 60-64, two aged 70-74 and a further two were 75-79. The remaining half of the group were aged over 80 years old (four were aged 80-84, and two were aged 85 or above). Perhaps as a result of this older demographic the health needs of this group were greater. One lived in sheltered housing, three stated that they had joint pain or mobility issues, and three others stated more complex health needs related to Parkinson’s, cancer and an autoimmune condition. The majority owned mobile phones, two of which had purchased big button mobiles with an emergency (SOS) button on the back. Three were using pendant alarms, one couple owned a blood pressure monitor and all were using computers/tablets and email.

### The devices

A number of available devices were introduced sequentially in six groups [8] and displayed in order to stimulate views and thoughts from the participants. Devices were chosen because they identified or aided common issues amongst older individuals, such as; fall detection often linked to location sensing, medication (pill) dispensing and vital sign monitoring. Prior to outlining each group of devices the participants were asked what they thought was the function of the device.

### Focus group data analysis

The groups were moderated using the same topic guide, with three groups of questions. The first covered what they knew about existing devices and their thoughts about barriers to use. The second section concerned issues of utility and cost of the devices which were on display and the third section covered discussion regarding a device in development. The focus group discussions were audio-recorded via a digital recording device (Philips DVT7000) with an omni-directional table-top microphone. Recording allowed for the discussion to be transcribed verbatim. The data obtained from the group transcripts were handled within NVivo 10 (a qualitative data analysis software package) and analysed using a thematic approach [9].

### The interviews

Preliminary findings from the user focus groups regarding their views on telehealthcare devices indicated the need to map the supply chain of these devices. A comprehensive understanding of other stakeholder views was sought in order to help explain why there are disconnections in terms of what users want. As a result, individuals who represented supply chain groups in this area were targeted. The relevant sample groups were: professional bodies, user groups, regulators, providers, county councils, charitable bodies, manufacturers and distributors, research funders and organisations, and trade associations. Those in senior management roles, such as Chief Executives or equivalent were contacted wherever possible, as experts in their fields, who would be willing to speak on behalf of their respective organisations. Suitable candidates were identified via extensive Internet searching and snowball sampling. Potential participants were invited to take part via email. To maintain anonymity those who participated have been placed into the sample groups outlined in Campling et al. [8].

### Interview guide

Interviews were conducted using a semi-structured guide covering the following areas:

- What are your views regarding the current uptake of telecare/telehealth devices?
- What are the barriers to device uptake?
- What are your views regarding user involvement in device development?
- What are your views on access and supply of devices?
- How can device uptake be promoted in the future?

The interviews ranged in length from between 30 minutes to an hour and a quarter. In total 27 interviews were conducted with experts; individuals were no longer approached once theoretical saturation had been achieved in the respective sample groups. Data collection and analysis occurred simultaneously so as to test recurrent themes arising from the analysis (constant comparative analysis [9]).

### Interview data analysis

All the interviews were audio-recorded and then transcribed verbatim. As with the data from the focus groups, the interview data were handled within NVivo 10 and analysed using a thematic approach [10]. Thematic analysis was selected as the method of analysis for the interview and focus group data as it enabled patterns (themes and resulting categories) across the two data sets to be constantly compared and drawn together to describe the experience of end-users in relation to the usability of telehealthcare devices. The analysis was performed through coding in phases to view meaningful patterns across the data. The phases of analysis were: familiarisation with the data, generation of initial codes, searching for themes amongst codes, reviewing of themes, appropriately defining and naming themes, and writing up findings.

## Results

Overall 22 categories emerged from the interview and focus group data. The barriers were pieced together across the categories to map telehealthcare device uptake in the UK (for further detail refer to [11]). Every barrier revealed by the thematic analysis will be outlined in turn.

i) The Lack of an Evidence Base
  Data from the expert sample exposed the lack of an evidence base underpinning telehealthcare devices and related services; this in turn created a substantial barrier to usage. With the ongoing climate of financial challenges in both health and social care it was recognised that “*it's quite difficult for commissioners to invest wholesale in something, which you know, really hasn’t got a massive evidence base behind it*” (Interviewee 005). Another participant stated: *“you've got this dreadful kind of barrier within health, if you haven't done a randomised controlled trial… then you can’t get it”* (Interviewee 013).
ii) The Buyer and the End-User
  The study revealed a profound distinction between those who selected and bought the devices and the ultimate end-users. This distinction may act as a disconnect to meeting end-user needs; affecting both the initial uptake and the on-going use of devices. For example family members often purchased devices for vulnerable relatives, which may have implications on usage. In one case a big button mobile phone with SOS button had been purchased by the focus group participant’s son, and the full functionality of the device was not understood as a result. Such purchasers are seeking to “*look after mum*”; purchasing products with the hope that the devices will be tried and accepted by their parents or close relatives.
iii) The Lack of End-User Awareness
  The focus groups demonstrated that the participants were totally unaware of the terms “telecare” or “telehealth”. This was something that the experts universally acknowledged as a further barrier to uptake. They recognised that the terminology in the area had evolved from initial industry distinctions between product areas for health and social care, to create unhelpful and even “*dangerous*” distinctions that “*damage the solution*” (Interviewee 023). An additional complication was that different definitions were used by the various players in the field. A number of experts called for the language used to be changed. The lack of success in gaining public awareness, and using appropriate language, meant that the focus group participants were not aware of the range of devices available.
iv) The Disconnect Between Health and Social Care
  The expert group highlighted the lack of integration between health and social care and the majority saw this as a barrier to device uptake, with important implications for end-users. As a result, there was a perceived lack of focus on preventive care needs. Those working within social care were keen to point out that within this division, where costs were spent in social care ultimately the savings were made in health care. The experts also recognised a reluctance on the part of end-users to engage with social services to access telecare devices. The fact that these devices are offered in a statutory setting was perceived to make many end-users uncomfortable. Arguably the social/health care divide equated to an equivalent divide between telecare and telehealth. As one expert said: “*I think there's room for health, clinicians and GPs in particular to be mindful of telecare and know what's available to be able to promote the use of telecare”* (Interviewee 020). Experts called for integrated working to break down these barriers, but it was recognised that this level of change could encounter conservatism and vested professional interests.
v) Complex Supply Routes
  For the experts, the complexity of the supply routes for telehealthcare products presented a barrier to device uptake which began with difficulties in the initial route to market. Interviewee 023 stated that the “*route to markets are complex in so far as they're not clearly defined”*, going on to argue that this was likely to evolve further with the greater role of retail pharmacies, with the products also changing as a result.
  Another expert articulated the complex routes of both device supply and service provision, stating that this led to fragmented service provision and the needs of end-users not being met. For all the experts the complexity was not just in the supply of the specific products, but included the back-up services.
  Commissioning of telehealthcare and related procurement of devices was key in explaining some of the complexity in supply. Within the UK this generally occurs through Framework Agreements on a four-yearly cycle. There are a number of procurement frameworks in place for telecare and telehealth equipment and services. However, in practice artificial divisions may be created, for example between assistive technology/“equipment” devices and telecare. Commissioning was a barrier to device uptake with the experts citing that commissioners “*don’t have enough guidance and support and advice as to how to actually commission these types of technologies, how to procure them and evaluate them successfully”* (Interviewee 014). Furthermore from an industry perspective Framework Agreements were a clear barrier to market entry, particularly for small-to-medium sized enterprises (SMEs) where expertise may not have developed in this regard.
vi) Access to Devices
  Difficulties in accessing devices were identified by all those who took part in the study. Focus group participants were uncertain about how to go about accessing and gaining supply of telehealthcare devices. They wanted somewhere to go to decide what might fit their needs and it was important that this process was not dominated by “*selling*” or “*commission*”. Those who spoke about accessing personal alarms through social services referred to the lack of choice available to them and also noted a reticence to engage with social services for fear of “*red tape*”, difficulties navigating the health/social care divide and in finding individuals within social services with the requisite knowledge of devices. Access was also hindered by cost issues, especially in view of the way on-going downward pressure on Local Authority budgets has impacted telecare services supplied via social services. Setting costs and eligibility criteria at the local level had led to wide pricing variation in practice, affecting device uptake. The implications of increased charging, particularly where telecare services may have been free in the past, had clear implications for device use. A participant said: “*I know in (region name) when it was free, when they started charging a lot of people did just say ‘take it out’*” (Interviewee 010). Both the experts and the users called for charges related to devices to be examined. Cost was also a concern associated with telehealth, with its relative expense preventing uptake. The cost burden in this case was at the health service level rather than for end-users. The existence of the NHS in the UK complicated matters in that health-related services are perceived as something that should be provided free to patients. For focus group participants it was clear-cut. Any telehealth system that could save other health-related costs, such as in-patient bed days, should be provided via the NHS. From an industry perspective the future commercial incentive for supply of devices hinged upon the ability to convince clinicians, particularly GPs, to supply such systems to individuals.
  Focus group participants universally condemned device purchase costs (whilst recognising that most would not need to be purchased) and some subscription charges. In the case of mobile (out of the home) personal alarms the subscription charges horrified the participants. Despite these criticisms, the experts perceived access to and supply of devices as something that would require greater flexibility. They interpreted the private purchase model as likely to develop much further in the future.
vii) The Difficulties in Recognition of Need
  Focus group participants recognised the difficulties inherent in identifying the point when an individual should start using a particular telehealthcare device. This was challenging, subjective and individual, often requiring a particular event, such as a fall, to trigger device uptake. They acknowledged that the identification of a suitable point in an illness trajectory for device introduction was fraught with difficulties and could hinge on an initial diagnosis and on-going assessment. There were particular difficulties in long-term condition management where changes occur slowly over long periods making recognition of changing needs at the individual level problematic.
  Most experts spoke of devices being offered too late and therefore not providing “*a significant impact in terms of prevention*” (Interviewee 013). One participant spoke of the concept of a “*golden period of time*” in which to introduce telehealth devices and systems thought to be when individuals have developed one or two conditions and need a period of education (Interviewee 005). Despite this concept of a “*golden period*” for device introduction many experts spoke of telehealthcare devices, particularly telecare, being decided upon and introduced too late. Telecare was seen as “*always put in in a crisis*” such as following hospital admission and to enable discharge “*so that the older person or whoever it's gone into, doesn’t really have much of a choice”* (Interviewee 004). Many also referred to the lack of informed choice by the individual because of this almost enforced usage following crisis situations.
  One interviewee added that potentially “*bleak*” thoughts associated with ageing and recognition of needs caused most to avoid considering their possible individual futures. As a result of this “*denial*” the experts often spoke about the need for high quality regular assessments of individuals by professionals. Focus group participants however did not specifically refer to professional assessment, but acknowledged that they might be unlikely to recognise their own unmet needs. The experts raised the problematic issues related to assessment of need which hinged upon accurate assessments. They were critical of the lack of expertise and professionalism from health and social care professionals in the area of telehealthcare devices and thought this was exacerbated by personnel turnover, of those “*who looked after the telecare grants in 2006/2008… five per cent or less of them are now in post*” (Interviewee 001).
viii) Attitudes of Health and Care Professionals Towards Devices
  Data from both focus groups suggested a concern regarding the attitude of health and care workers towards the use of devices, which in turn acted as an additional barrier to device usage. The experts stressed the need for a high level of clinical buy-in in terms of promoting the usage of telehealth devices. Without this buy-in telehealth services would fail. Resistance of professionals to devices was a key barrier uncovered in the data and was reported by the majority of the expert group. This was seen as coming from medics and nurses. Clinician resistance was key to telehealth uptake, but resistance was also something that had been seen on the part of social care professionals. Resistance was believed to be predominantly related to increasing workload associated with changing working practices. Within the pressurised health services there was a pay-off that required elements of existing services to be dropped to establish new telehealth services. Alternatively if extra staff were drafted in, this was seen as perpetuating the barriers as separate systems were established, rather than establishing these devices and systems as part of routine in-practice care.
ix) Mistrust Between Player Groups
  The study data revealed mistrust between the respective player groups. Industry representatives argued that they had to work extremely hard to build trust within both health and social care sectors. The provision of services and devices was seen as hard won and market entry as challenging: “*they don’t trust you to come in and run this service for them… it takes a long time to build that trust up*” (Interviewee 003). Mistrust was evident on all sides, and those participants working outside of the telehealthcare industry could also be mistrustful of companies. This was related to the market dominance of the larger telehealthcare companies, which in turn was interpreted as preventing product development, with insufficient incentives to develop new products as sufficient volumes of existing products are being sold (Interviewee 026).
x) Local Variation and Inappropriate Product Usage
  Profound local, regional and national variation between product usage and supply were exposed, as well as the additional but associated issue of inappropriate product supply and use. At a local level, variation in practice occurred not only because of fragmented decision-making and purchasing power, but also because of variation in assessment. This may be exacerbated by variation in assessor training, whether by companies or social care departments within local authorities. Consequently, variation in who holds the decision-making power, plus those in charging structures and training, leads to variation in product recommendations. As a result recommendations may be inappropriate as the generation of expertise can be stifled where these differences exist.
xi) “Big Data”
  Issues of valid data generation and the related issues of data security, confidentiality and transfer were ones raised by experts and end-users alike. Focus group participants raised concern over some telehealthcare devices and their systems. Regarding telehealth devices, the focus groups raised issues regarding data transfer and confidentiality. For them this was crucial because of the unwitting implications such as for health insurance. They went on to discuss specifics, concluding that data should be accorded the same level of confidentiality as by the NHS. They were more concerned that data should be viewed and assessed; the issue not being security per se, but the active and appropriate use of data. The consensus was that data confidentiality was less of a concern providing there were tangible health benefits, timely data transfer and sharing, which outweighed possible disadvantages. Nonetheless, they recognised the existing lack of NHS facilities for the timely data transfer of telehealth data. An expert participant noted that the interface between health and social care systems was poor, at best; hence data was not transferred appropriately relating to individual user needs. The experts had greater concerns regarding data security than the focus group participants. They felt that although there were “*big issues about IG (information governance) and how secure it’s got to be*” there was often an inference that this was perhaps more of a barrier that it should be: “*we can get really hung up on that*” (Interviewee 018). Despite these differences there was universal agreement that the transfer and use of the data was vital. Additionally for businesses, the lack of feedback from the health and care sectors regarding user need, constituted profound barriers to product and service development. This lack of use of available data, even where it exists, meant end-user needs were not being met. As a result another expert called for monitoring centres to become hubs in a complex telehealthcare information network.
xii) The Response is Key
  For all participants the response provision for devices was critical. Devices were a mechanism for accessing a system and services; and for the focus group participants the central monitoring services behind the devices were vital. There was a reluctance to involve personal contacts such as neighbours and family, with a recognition that the systems were only as good as the contacts provided. Therefore because of these difficulties, monitoring centres and responder services were viewed as an important requirement. The logistics of any monitoring centre behind a device were another concern for the focus group participants. For reassurance they wanted an efficient and prompt response that was “*friendly and supportive*” (Bedfordshire focus group participant). Furthermore, they wanted a response that was “*personalised to individual requirements*” (Oxfordshire focus group participant). The experts also highlighted the importance of the services and systems behind the devices: “*the monitoring needs to happen 24/7, especially in terms of telecare. It's an emergency system so it needs to be 24/7 monitoring*” (Interviewee 003). A focus was placed on the services that the devices access rather than the devices themselves: “*we should be talking about the services rather than the technologies*” (Interviewee 008). Thus, although the experts clearly identified the need to focus on back up services, they identified numerous functional shortcomings. This could be related to the monitoring centres or the lack of comprehensive responder services. Monitoring centres were seen to be in a “state of flux” with a drive towards consolidation.
xiii) A Lack of Expertise
  Both focus group participants and experts recognised the lack of expertise and knowledge regarding the range of telehealthcare devices. Even for those working in the sector it was seen as confusing, preventing expertise from developing because of fragmented services and variation in practice. For example, home-care workers were identified as knowledgeable on the needs of their clients, but lacking knowledge of devices. Training provision for such groups was a complicated issue as Local Authorities frequently contract out care provision. Thus uniformity of training across numerous private care providers was problematic. In addition, it was recognised that home care workers have “*very limited time, limited resources, a high staff turnover, how do you make sure everyone’s up to speed?”* (Interviewee 010).
  Training was discussed by the expert sample, where it was seen as a major barrier to device uptake. Unless receiving training, health and care professionals are reliant on gaining experiential knowledge through practice. Those involved day-to-day in assessment and the prescribing of devices will develop knowledge and confidence in the products and what will meet specific needs. Without this, “*they'll lose confidence and they never then go on to prescribe telecare*” (Interviewee 017).
  The focus group participants called for independent experts to be able to advise them, whether telehealthcare specialists, occupational therapists, social services or community nurses with sufficient expertise in the field. Many had experienced the “*red tape*” of navigating social services and difficulties in “*finding the right person who’s going to have the knowledge*” (Oxfordshire focus group participant). To this end, they recognised that there were often high street “*mobility centres*” providing larger assistive technology items and called for similar places to try out telehealthcare devices, offering somewhere for them to view the devices in person. This lack of independent shops to explore and test devices was also acknowledged by the expert sample, but they noted some health centres and public spaces were being used for the purpose. The expert participants called for independent web-based reviews of products. Internet forums featuring product reviews were seen as a valuable mechanism for sharing what “*equipment has meant*” to an individual as no one size fits all (Interviewee 012). The need for an “*honest broker*” that could “*give people an independent source of information on the whole market*” regarding “*all the products that are out there*” was viewed as vital (Interviewee 017). Some Internet resources were identified, however the current lack of comprehensive and freely available material accessible by both end-users and professionals was recognised. One expert identified Telehealthcare specialists as a means of addressing the lack of expertise in the area, calling for their widespread introduction to comprehensively advise clinicians within CCGs. Such specialists, once in place, could bridge the gap between clinicians and suppliers, as at present most clinicians will not agree to meet a supplier/manufacturer if approached. Furthermore, the experts also identified the need for specialists to advise commissioners in this field, particularly between the variations in services offered (Interviewees 014 and 024).

## Discussion

This study identifies critical issues in uptake of telehealthcare devices and the consequent barriers to their adoption. Often, the trigger for purchase is the diagnosis of an illness or an emergency event such as a fall, leading to increased feelings of vulnerability, loss of control or possibly dependence on others. Such prompts do not predispose to well researched purchase or usage. Whilst there are several companies with competing products, we like others found that understanding and awareness of the products and their potential benefits was not always fully comprehended by users. Another consequence we identified was that when devices were bought by younger relatives or carers [12] this often lead to a lack of awareness and engagement by the end user.

Purchasing was a key issue and previous work has shown that a mixed supply market, where devices can be obtained from health or social care services or purchased directly from manufacturers has arisen due to an inability of healthcare organisations to meet the needs of the client [13]. One issue with such a mixed supply approach has been a lack of interoperability of the devices with other systems [6] something which we also highlighted. Moreover our results suggest that the absence of either a solid independent evidence base or accessible independent expertise resulted in a lack of confidence of users in the area of device choice. This may be a consequence of a market which has yet to reach maturity and for which there is little evidence of the return on investment required [6]. Indeed up-front costs of technology in a tight financial climate may increase the risks of waste and blame if the technology is not used or is not seen as a success [14]. Partly this may be due to the approach; the assessment methodologies and criteria for telehealthcare devices require the existing paradigm of the pharmaceutical model using randomised controlled trials (RCTs) to be carefully scrutinised. This may be an unsuitable model for telehealthcare research where the product development cycle can be fast paced with rapid evolution over short timescales such as ICT based services which themselves evolve in practice.

In addition a lack of maturity in the market has introduced complexity associated with whether the end user was the purchaser or whether it was purchased by an intermediary who may or may not have been the installer. Split responsibilities for the upkeep of the system and the monitoring of data have in some cases contributed to the disconnection between health and social care workers. Different funding streams between health and social care and some resistance from the professionals in these areas have also hindered acceptance [15, 16]. This resistance among care workers and professionals can act as a barrier to successful implementation; carers and care workers have expressed uncertainty and anxiety about the use of telecare and concerns about not having the necessary skills, which relates to a lack of knowledge and training in how to use these technologies [17]. Therefore training courses for carers and care workers could be important in reducing anxieties about these technologies, and providing advice at the point of installation may be particularly key. As in our study, the AKTIVE Consortium [17] found a lack of information and awareness; with carers and care workers often unaware of the range of telecare services available and how to access them. To address this more needs to be done to raise awareness, also integrating telecare with effective face-to-face services and support, including effective assessment of needs.

This study has limitations associated with the small sample size; the focus groups were derived from two geographical distinct areas and our experts were derived from those available and willing to participate. The small sample size for end-users (focus group participants) limits our ability to generalise from the data. However the focus groups themselves were relatively large to account for non-attendance in the older population, but only one participant did not attend on the day. Increasing the number within a focus group can improve the opportunities for obtaining a representative view to aid transferability of findings, but equally it may limit the active contribution of some participants. Additionally, the fact that the focus groups participants were self-selecting may also limit the implications of our findings. We incorporated the views of experts to ameliorate these limitations but were also aware that some of these limitations (small sample size, selection bias arising from availability to participate) could equally also apply to this sample. Nevertheless we believe our data exemplifies common barriers to the uptake of devices which impact on their usage.

Recent reorganisation of the NHS to channel funding to areas of primary care led to the formation of clinical commissioning groups who receive over 60% of the budget [18]. Public health, once the responsibility of the NHS has now been reallocated to Public Health England in addition to local authorities. The presence of multiple autonomous organisations within a national framework, the diversity around the country and the different approaches to implementation [14] have not always been conducive to effective management of innovation and the introduction of telehealth; and this was underscored by our study over issues surrounding the difficulties around interaction of health and social care bodies. Such difficulties of providing effective communication in a fragmented care system have been previously identified especially in the area of telehealth.

## Conclusion

This study has identified a number of key areas as barriers to telehealthcare uptake. The first is the lack of a robust evidence base for the effectiveness of telehealthcare devices. Without this decisions will be made that fail to meet the needs of users and future product development will be delayed as companies looking to invest in new designs or seeking to enter the market have little data on which to base potential commercial propositions. The second area is a lack of independent expertise and knowledge regarding telehealthcare products, particularly by both health and care professionals. Third, our study demonstrated a lack of public awareness of telehealthcare devices, something further complicated by the lack of understanding of the terms used in the field. Finally, any future drive to develop assessment methodologies and criteria for telehealthcare device approval requires the existing paradigm of the randomised controlled trial to be carefully scrutinised. The question of whether RCTs are fit for purpose for device evaluation is debatable.

Future research in this area needs to concentrate on providing acceptable alternative methodologies, as valid alternatives to RCTs especially when health and care budgets are increasingly being squeezed. The challenge is in developing and using technology that can respond successfully to the full range of individual needs, in a way that promotes personalised care.

## Abbreviations

ICT: Information and Communication Technology
RCT: Randomised Controlled Trial
SME: Small to Medium Enterprise
TECS: Technology Enabled Care Services
WSD: Whole System Demonstrator.
